# Gastroduodenal Perforation in Cancer Patients: Association with Chemotherapy and Prognosis

**DOI:** 10.3390/medsci11020026

**Published:** 2023-03-28

**Authors:** Melissa Mello Mazepa, Marina Alessandra Pereira, Arthur Youssif Mota Arabi, André Roncon Dias, Ulysses Ribeiro, Bruno Zilberstein, Luiz Augusto Carneiro D’Albuquerque, Marcus Fernando Kodama Pertille Ramos

**Affiliations:** Instituto do Cancer, Hospital das Clinicas HCFMUSP, Faculdade de Medicina, Universidade de Sao Paulo, Sao Paulo 01246-000, Brazil; melissa.mazepa@hc.fm.usp.br (M.M.M.); arthur.arabi@hc.fm.usp.br (A.Y.M.A.); andre.dias@hc.fm.usp.br (A.R.D.); ulysses.ribeiro@hc.fm.usp.br (U.R.J.); bruno.zilberstein@hc.fm.usp.br (B.Z.); luiz.carneiro@hc.fm.usp.br (L.A.C.D.)

**Keywords:** neoplasms, spontaneous perforation, peptic ulcer perforation, chemotherapies, preoperative complications

## Abstract

Background: Gastroduodenal perforation stands out as one of the complications in cancer patients. Despite its high mortality, its characteristics are still poorly described. This study aimed to evaluate the characteristics and outcomes of cancer patients who had gastroduodenal perforation, and the influence of chemotherapy (CMT) in these cases. Method: A retrospective analysis of patients who underwent emergency surgery with an intraoperative finding of gastroduodenal perforation. Patients who performed CMT within 60 days before perforation were considered as the CMT group. Results: Among 45 patients included, 16 (35.5%) were classified as the CMT group and the remaining 29 (64.5%) patients as the non-CMT group. There was no difference between the groups regarding sex, age, BMI, comorbidity, and laboratory exams. ECOG 2-3 was significantly more frequent in the CMT group (68.8% vs. 34.5% *p* = 0.027). Major postoperative complications were similar between both groups (75% vs. 58.6%, *p* = 0.272). The sepsis of abdominal focus was the main postoperative complication. The 30-day mortality was 55.6%, with no difference between non-CMT and CMT groups (62.5% vs. 51.7%, respectively; *p* = 0.486). A multivariate analysis of risk factors showed that only an age of ≥65 years was related to 30-day mortality. Conclusions: Patients with gastroduodenal perforation and oncologic treatment present high mortality, regardless of receiving recent CMT.

## 1. Introduction

The progressive aging of the population in several countries has turned cancer into an important cause of morbidity and mortality. It is estimated that cancer is currently the second leading cause of death in the world [1]. During its treatment, complications may occur due to the natural evolution of the tumor, the decompensation of pre-existing diseases, and the treatment used [2]. Among the complications related to the digestive tract, the perforation of hollow viscera is a surgical emergency with high lethality.

In the general population, gastroduodenal perforation can occur as a complication of pre-existing peptic ulcer disease (PUD). In this scenario, the morbidity and mortality are already well documented. The lifetime risk of perforation is approximately 2–10% in cases of untreated PUD, and despite an improvement in resuscitation, and intensive and surgical care, the mortality rate remains around 25% [3,4].

In cancer patients, gastroduodenal perforation can be spontaneous and associated with tumors located in other organs. In this setting, the possible influence of chemotherapy (CMT) and drug treatment on its pathogenesis and prognosis has been discussed [5,6,7]. The toxicity of some CMT and anti-angiogenic agents can affect the mucous membranes of the entire gastrointestinal tract, with stomatitis and diarrhea being the most common complications and perforation the most critical [5,6,8,9].

There are very few case series reporting the outcomes and prognostic factors of oncology patients with gastroduodenal perforation undergoing emergency surgery. Maeda et al. found that patients older than 70 years, the Eastern Cooperative Oncology Group (ECOG) 1–2, and hypoalbuminemia are factors associated with in-hospital death in cancer patients undergoing emergency surgery for a perforated acute abdomen but without specifying the perforation sites in the gastrointestinal tract [10]. Therefore, the present study aimed to evaluate the characteristics and survival outcomes of cancer patients who had gastroduodenal perforation unrelated to the primary tumor during treatment in a reference cancer center, and its relation to the CMT.

## 2. Materials and Methods

Data from all patients who underwent emergency surgery for acute abdomen at our Institution from February 2010 to March 2021 were retrospectively analyzed. Patient data were obtained through an electronic medical record. Patients with clinical suspicion of perforating acute abdomen after performing a computed tomography of the abdomen who underwent a surgical procedure were evaluated [11,12]. After, only patients with intraoperative findings of gastroduodenal perforation in the surgical report were included. Patients with pathological findings of perforated gastric tumors were excluded, as well as perforations in other locations of the digestive tract.

Clinical characteristics and laboratory tests were considered based on the last medical appointment before hospitalization for the emergency surgery. Clinical characteristics included sex, age, body mass index (BMI), a history of peptic ulcer disease (PUD), the Eastern Cooperative Oncology Group (ECOG) scale, and the Charlson–Deyo comorbidity index (CCI), without considering age and neoplasm as comorbidities [13]. Laboratory exams included hemoglobin levels (g/dL), albumin (g/dL), and neutrophil-to-lymphocyte ratio (NLR). Smoking status was classified as current smokers and non-smokers. Former smokers who quit smoking in the last 2 years were considered along with current smokers. The actual use of other medicaments associated with risk gastroduodenal perforation as non-steroidal anti-inflammatory drugs (NSAIDs) and oral corticosteroids and protective proton pump inhibitors (PPIs) was also evaluated. The use of drug data was collected from active prescriptions before surgical admission.

For analysis, patients were divided into two groups to assess the association of CMT with surgical outcomes. Patients were considered as having active treatment when performed within 60 days before the perforation (CMT Group) and inactive if the last cycle of treatment was over 60 days (non-CMT-group). The CMT schemes include platinum, fluorouracil, and taxane-based regimens. Previous radiotherapy was reported regardless of the time interval related to the perforation. 

The site of the perforation and the type of surgical procedure were evaluated. Postoperative complications (POC) were graded according to the Clavien–Dindo classification [14]. The main outcome evaluated was mortality at 30 days after surgery. Mortality at 90 days was also examined.

Statistical analysis was performed using SPSS software version 20.0 (SPSS, Chicago, IL, USA). The chi-square test or Fisher’s exact test was used for categorical variables, and the *t*-test or Mann–Whitney test was used for continuous variables. Binary logistic regression, with the odds ratio and the respective 95% confidence intervals (95%CI), was used to evaluate the risk factors for 30-day mortality. Survival was estimated by the Kaplan–Meier method, and the curves were compared using the log-rank test. Survival was considered, in months, from the date of surgery to the date of death, or the last medical appointment. Results were considered significant when *p* < 0.05.

## 3. Results

**Figure 1:**
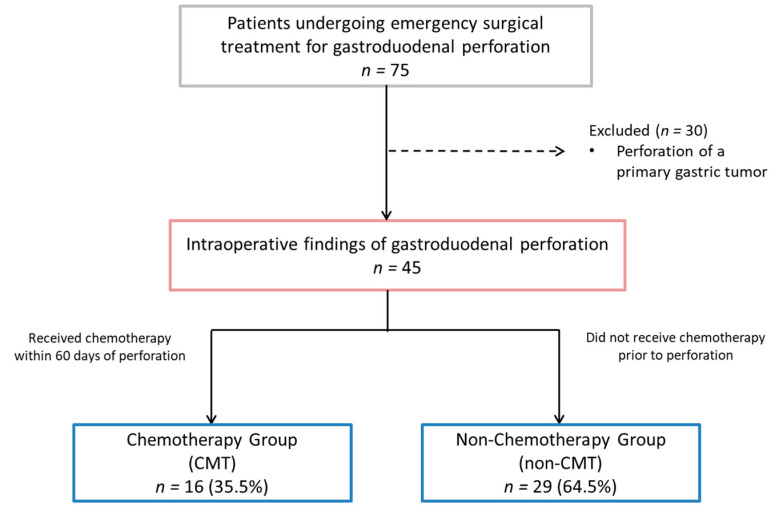
Study flowchart.

During the analyzed period, 75 patients who underwent emergency surgical treatment for gastroduodenal perforation were identified. Thirty patients who had a perforation of a primary gastric tumor were excluded from the analysis. The remaining 45 patients were included in the final analysis (Figure 1). 

The mean age of patients was 64.4 years, most patients were male (71.1%), and the mean BMI was 21.8 kg/m² (SD4.6). Most patients had primary tumors in the gastrointestinal tract (26.7%), followed by the head and neck (24.4%). Regarding laboratory tests, the mean levels of hemoglobin and serum albumin were 11 g/dL (SD 2.1) and 3.2 g/dL (SD 0.7), respectively.

Active CMT was verified in 16 patients (35.5%) and was considered as the “CMT group”. The remaining 29 patients (64.5%) who did not receive CMT in the 60 days before the perforation constituted the “non-CMT group”. 

The initial clinical characteristics of both groups are summarized in Table 1. Patients in the CMT group had worse ECOG grades than the non-CMT group (*p* = 0.027). There were no differences regarding sex, age, BMI, comorbidities, NLR, albumin, and hemoglobin levels between CMT and non-CMT groups. Most patients had no history of PUD. 

No difference was found between the groups regarding the site of the primary tumor (*p* = 0.605) and the presence of metastasis. There was also no difference in the use of NSAIDs and corticoids, and PPIs (Table 1).

Regarding the surgical characteristics of the perforation, 26 patients (57.8%) had gastric and 19 had duodenal (42.2%) perforations. The most performed surgical technique was suture with omentum patch, in 36 patients (82.3%), followed by partial gastrectomy in 8 patients (17.7%). There was no difference in surgical characteristics and postoperative outcomes between CMT and non-CMT groups (Table 2). The mean length of hospital stay was 11.1 days (± 9.84), with a median of 8 days (IQR 3–15.5). 

The 30-day mortality rate of patients with gastroduodenal perforation was 55.6%, with no statistical difference between the groups (62.5% vs. 51.7%, *p* = 0.487). The 90-day mortality rate for all patients was 71.1%, without a difference between groups (75% vs. 69%, *p* = 0.669) (Table 2). 

The type and grade of POC are shown in Table 3. Postoperative abdominal infection with sepsis was the most common cause of mortality (Clavien V).

In multivariate analysis for risk factors related to 30-day mortality, age ≥ 65 years was the only independent factor associated with 30-day mortality (OR = 3.86, 95%CI: 1.01–14.84, *p* = 0.049). (Table 4). Chemotherapy was not associated with an increased risk of 30-day mortality (OR = 1.56, 95%CI: 0.45–5.41, *p* = 0.487).

**Figure 2:**
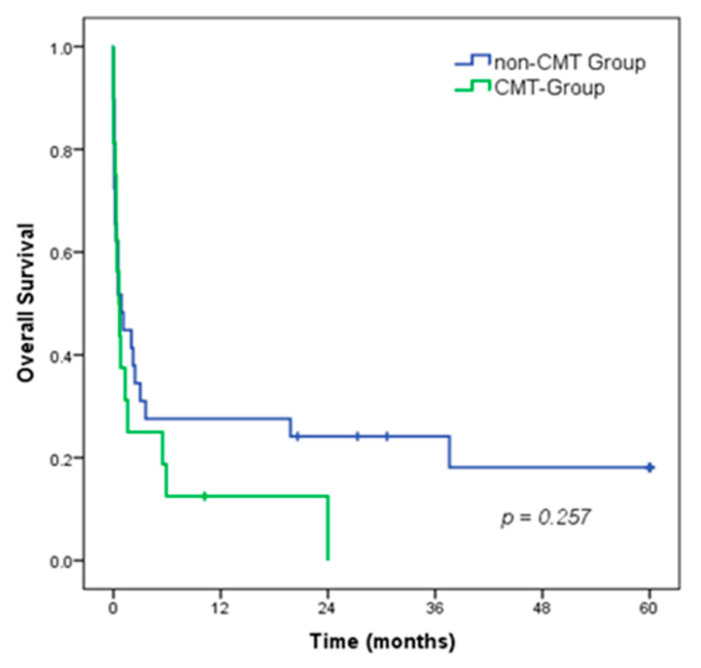
Overall survival of patients with gastroduodenal perforation, according to the CMT groups.

The 5-year overall survival (OS) rate of all patients was 12.7%, with a median survival of 0.7 months. There was no difference in OS between patients in the CMT group compared to the non-CMT group (median OS of 0.6 vs. 0.9 months, respectively; *p* = 0.257). (Figure 2). 

## 4. Discussion

During cancer treatment, surgical emergencies unrelated to the primary tumor may occur. Among these, the perforated acute abdomen stands out due to its severity and the immediate need for a surgical evaluation. Although there is no direct relationship with the primary tumor, the question of whether the treatment employed may be involved in causing the perforation and impacting the prognosis is recurrent. To clarify this issue, the present study analyzed the characteristics of cancer patients who had gastroduodenal perforation with or without recent CMT, and risk factors associated with mortality in this population. In both groups, mortality was quite high, above 50%, but was not associated with a higher risk of 30-day mortality.

In the present study, 35.5% of patients with perforations received CMT, with included different regimens. Although monoclonal antibodies and antiangiogenic therapies are associated with DUP and gastrointestinal tract perforation [9,15,16,17], none of the patients in our study received these drugs. Spontaneous perforation of the GI tract after initiation of chemotherapy with drugs such as fluorouracil with cisplatin has been reported [6] Unfortunately, the administration of different regimens in our cohort, associated with the relatively small number of cases, did not allow us to verify the association of some specific regimens with the occurrence of perforation. 

Remarkably, the rate of current smokers in our cohort was much higher (71.1%) than the previous report of 9.1% of active smokers in the Brazilian population [18]. The more advanced age of the patients included in the study—associated with the fact that the decrease in the smoking habit is more recent—may partially justify this difference. In addition, smoking is a carcinogenesis factor of different primary sites, mainly in the upper respiratory and digestive tract, which accounts for just over half of the cases in this study [19,20,21]. In Brazil, it was found that over 70% of patients with head and neck cancer, and 90.5% of patients with lung cancer were active smokers [22,23]. Indeed, smoking constitutes a risk factor for PUD as it promotes increased gastric secretion and local oxidation, in addition to reducing angiogenesis and mucosal regeneration [24]. In the present study, the history of previous PUD was verified in approximately 20% of cases. This rate was higher than the estimated lifetime prevalence of around 5–10% in the general population [25]. Therefore, the aforementioned higher prevalence of smoking may be related to this result.

Noteworthily, more than half of the patients in the study had metastatic tumors. Metastasis is a complex systemic disease that develops because of interactions between tumor cells and their local and distant microenvironments [26]. Factors secreted by cancer or stromal cells in the primary tumor can act in distant organs in a way that promotes metastasis. This action may affect the physiology of other systems, and its participation as a causal factor for gastrointestinal tract perforation should be considered.

Regarding the use of other drugs that can be associated with perforation [27], we found a low frequency of NSAIDs use (4.4%). Although the frequency of patients who used corticosteroids was higher (37.8%), the number of users of PPI was not much lower (31.1%). This characteristic may reflect the concern of the medical care team with the risk of perforation [28,29]. 

Concerning the surgical technique employed, perforation suturing was the most used technique. Although less effective in the long term, the severity of the patients, associated with the poor prognosis due to the presence of metastases, fully justifies this option [7]. After local control of the perforation by surgery, the sepsis treatment protocol must be started. Besides early diagnosis and minimal surgical delay, it includes volume resuscitation, antibiotic therapy, and adequate nutrition postoperatively. Early implementation of the protocol can reduce mortality from 27% to 17% in the non-oncologic population, and this approach should be pursued even more rigorously in oncologic patients [3,30,31]. In our study, the mean postoperative hospital stay was 11.1 days. Although lower than expected, this value should not be associated with a quick recovery. The high 30-day mortality rate may have distorted this result with early deaths. As a comparison, we previously reported a length of stay of 12.5 days, with a 30-day mortality of 7.9% for symptomatic gastric cancer patients in clinical stage IV undergoing palliative gastrectomy [32].

Certainly, the high 30-day mortality rate of 55.6% is higher when compared to reports for non-oncology patients, which ranges from 10–30% [3,33]. The mortality rates draw further attention when increasing the analysis period to 90 days (71.1%), compared to reports of 20–30% of non-oncology patients [4]. The 30-day mortality found in this study was also higher than described for patients under CMT with perforation of other gastrointestinal hollow viscera, reported in about 44% [5,10]. Advanced age, active oncologic disease, and hypoalbuminemia are some risk factors generally associated with mortality after gastroduodenal perforation [4,19]. In the present study, the included population consisted precisely of oncology patients. Further, the patients already had characteristics of worse prognosis, such as lower hemoglobin and albumin levels, and metastatic disease, which made it difficult to identify factors for early mortality.

In elderly cancer patients undergoing elective surgery, age is a factor associated with higher postoperative complications, length of hospital stay, and mortality [34]. The higher mortality in elderly patients can be understood by the frailty and lower physiological responsiveness that accompanies aging, making it more difficult for these patients to survive a severe insult, such as acute abdomen perforation [30]. The analysis of the severity of complications in the present study showed a high proportion of Clavien V cases, among the major complications demonstrating a high proportion of cases with failure-to-rescue. As expected, the main cause of mortality was abdominal infection with sepsis. 

This study has some limitations that should be raised. This is a single-center retrospective study, and the small patient cohort limits some analyses. The group of patients studied was heterogeneous with different primary tumors and CMT regimens. Since we only analyzed the patients who had perforation, the identification of causal or risk factors is compromised by the absence of data from the entire population at risk of the event. This fact also made it impossible to verify if there is a higher frequency of perforation in oncology patients compared to the general population. 

In terms of strengths, this study covered a long period, including all patients who underwent surgery for gastroduodenal perforation. Due to the decrease in perforations with the use of the PPIs, its reports and treatment experience have diminished in the more recent literature. Because it is also a clinical situation frequently performed in patients already in palliative care, its outcomes have been seldom reported. Accordingly, the results of the present study reinforce the special scenario of cancer patients who specifically present gastroduodenal perforation and its high mortality rate.

## 5. Conclusions

Gastroduodenal perforation occurring in cancer patients had unfavorable short-term outcomes, regardless of recent chemotherapy use, with a 30-day mortality rate greater than 50%. Advanced age was the only independent risk factor associated with mortality, and postoperative abdominal infection with sepsis was the most common cause of POC. Thus, due to the dismal prognosis, the early diagnosis of perforation with surgical intervention remains essential to improve survival, and older age patients with emergency abdominal surgery may be at increased risk of serious and life-threatening conditions due to already unfavorable clinical status and medical comorbidities. 

## Figures and Tables

**Table 1:** Initial clinical characteristics, tumor status, and associated medical treatments of patients—CMT and non-CMT groups.

Variables		CMT Group	Non-CMT Group	*p*
		*n* = 16 (%)	*n* = 29 (%)	
Sex				0.169
	Female	7 (43.8)	6 (20.7)	
	Male	9 (56.2)	23 (79.3)	
Age				0.218
	Mean (SD)	61.9 (9.3)	65.9 (10.6)	
Body mass index				0.245
	Mean (SD)	22.9 (5.9)	21.2 (3.7)	
Charlson–Deyo comorbidity index				0.494
	0–1	10 (62.5)	22 (75.9)	
	≥2	6 (37.5)	7 (24.1)	
ECOG scale				**0.027**
	0–1	5 (31.2)	19 (65.5)	
	2–3	11 (68.8)	10 (34.5)	
Smoking status				0.491
	No	3 (18.8)	9 (31)	
	Current smoker	12 (81.2)	20 (69)	
History of peptic ulcer disease				1.000
	No	13 (81.2)	23 (79.3)	
	Yes	3 (18.8)	6 (20.7)	
Hemoglobin (g/dL)				0.704
	Mean (SD)	10.8 (2.0)	11.1 (2.3)	
Albumin (g/dL)				0.282
	Mean (SD)	3.4 (0.7)	3.1 (0.7)	
Neutrophil-to-lymphocyte ratio (NLR)				0.174
	Mean (SD)	10.15 (8.89)	6.73 (4.79)	
Primary tumor				0.605
	Head and Neck	2 (12.5)	9 (31)	
	Gastrointestinal Tract	5 (31.2)	7 (24.1)	
	Thorax	4 (25)	3 (10.3)	
	Gynecology/breast	1 (6.2)	2 (6.9)	
	Urological	3 (18.8)	7 (24.1)	
	Others	1(6.2)	1 (3.4)	
Oncological status				0.360
	Absent/Localized	6 (37.5)	15 (51.7)	
	Metastatic	10 (62.5)	14 (48.3)	
Previous radiotherapy				0.771
	No	9 (56.2)	15 (51.7)	
	Yes	7 (43.8)	14 (48.3)	
Use of NSAIDs				0.531
	No	16 (100)	27 (93.1)	
	Yes	0 (0)	2 (6.9)	
Use of PPIs				0.197
	No	9 (56.2)	22 (75.9)	
	Yes	7 (43.8)	7 (24.1)	
Corticosteroid Use				0.209
	No	8 (50)	20 (69)	
	Yes	8 (50)	9 (31)	

NSAIDs, non-steroidal anti-inflammatory drugs; PPI, proton pump inhibitor. *p*-values in bold were statistically significant.

**Table 2:** Surgical data and outcomes of patients—CMT and non-CMT groups.

Variables		CMT Group	Non-CMT	*p*
		*n* = 16 (%)	*n* = 29 (%)	
Location of perforation				0.878
	Duodenum	12 (41.4)	7 (43.8)	
	Stomach	17 (58.6)	9 (56.2)	
Surgical technique				0.427
	Suture	25 (86.2)	12 (75)	
	Partial gastrectomy	4 (13.8)	4 (25)	
Complications—*Clavien–Dindo*				0.272
	0-I-II	12 (41.4)	4 (25)	
	III-IV-V	17 (58.6)	12 (75)	
Anastomosis/suture fistula				0.166
	No	27 (93.1)	12 (75)	
	Yes	2 (6.9)	4 (25)	
30-day mortality				0.486
	No	14 (48.3)	6 (37.5)	
	Yes	15 (51.7)	10 (62.5)	
90-day mortality				0.669
	No	9 (31)	4 (25)	
	Yes	20 (69)	12 (75)	

**Table 3:** Postoperative complications (POC) and grade (*Clavien–Dindo*).

Complication/Grade	II	III	V
Cardiac	1	–	–
Urological	1	–	3
Pulmonary	–	–	2
Upper digestive hemorrhage	0	1	1
Abdominal wall dehiscence	0	1	0
Abdominal infection with sepsis	–	1	14
Total	2	5	24

**Table 4:** Univariate and multivariate analysis of factors associated with 30-day mortality.

	Univariate			Multivariate *		
Variables	OR	95% CI	*p*	OR	95% CI	*p*
Male (vs. Female)	0.44	0.11–1.74	0.245	–	–	–
Age ≥ 65 (vs. >65 years)	3.87	1.09–13.81	**0.037**	3.86	1.01–14.84	**0.049**
CCI ≥ 2 (vs. 0–1)	0.86	0.21–3.60	0.859	–	–	–
Hemoglobin ≤ 11 (vs. >11)	1.18	0.36–3.89	0.787	–	–	–
Albumin < 3.5 (vs. ≥3.5)	0.91	0.19–4.36	0.913	–	–	–
ECOG grades 2–3 (vs. 0–1)	2.36	0.70–7.94	0.164	2.15	0.52–8.95	0.293
Current smoker (vs. no)	0.53	0.13–2.11	0.369	–	–	–
Metastasis (vs. no)	2.67	0.79–8.95	0.112	1.66	0.41–6.73	0.479
Stomach (vs. duodenum)	1.23	0.37–4.03	0.736	–	–	–
Gastrectomy (vs. suture)	0.41	0.09–1.97	0.266	–	–	–
CMT (vs. non-CMT)	1.56	0.45–5.41	0.487	–	–	–

* included in the multivariate model variables with *p* < 0.200; OR: odds ratio; CI: confidence interval; and CMT, chemotherapy. *p*-values in bold were statistically significant.

## Data Availability

Not applicable.
